# The implementation of the Strategy Europe 2020 objectives in European Union countries: the concept analysis and statistical evaluation

**DOI:** 10.1007/s11135-016-0454-7

**Published:** 2016-11-15

**Authors:** Małgorzata Stec, Mariola Grzebyk

**Affiliations:** Faculty of Economics, University of Rzeszów, Cwiklinskiej 2 Str., 35-601 Rzeszow, Poland

**Keywords:** Development, Changes, Strategy Europe 2020, Classifications of countries, European Union

## Abstract

The European Union (EU), striving to create economic dominance on the global market, has prepared a comprehensive development programme, which initially was the Lisbon Strategy and then the Strategy Europe 2020. The attainment of the strategic goals included in the prospective development programmes shall transform the EU into the most competitive economy in the world based on knowledge. This paper presents a statistical evaluation of progress being made by EU member states in meeting Europe 2020. For the basis of the assessment, the authors proposed a general synthetic measure in dynamic terms, which allows to objectively compare EU member states by 10 major statistical indicators. The results indicate that most of EU countries show average progress in realisation of Europe’s development programme which may suggest that the goals may not be achieved in the prescribed time. It is particularly important to monitor the implementation of Europe 2020 to arrive at the right decisions which will guarantee the accomplishment of the EU’s development strategy.

## Introduction

Globalization, growing international competitiveness, dynamic development of information and communications technology, growing role of knowledge and innovation, demographic problems and shortage of resources, at the turn of the nineteenth and twentieth centuries, created great challenges for the European Union (EU). Managing these tasks resulted in passing the so-called Lisbon Strategy in 2000.

The pursuit of this strategy was to raise the EU competitive power on the global market but concurrently to retain the social values essential for the European citizens. As a general objective, it was assumed that the EU would be transformed by 2010 into the most competitive and dynamic economy in the world based on knowledge, develop in conditions of sustainable economic growth and provide greater employment and social cohesion (Capello and Lenzi 2013; Kasza 2009; Steurer and Hametner 2013; Sulmicka 2016).

From the start of the Union, it was not intended to build Europe at a single stroke, but by setting in motion a true solidarity among the countries through concrete actions to reduce asymmetries among the European Union’s (EU’s) countries in order to increase social and economic cohesion within its borders (Bhabha 1998; Goncalves 2011; Grzebyk and Stec 2015; Marrocu and Paci 2013; Martin et al. 2012; Roger 2014).

However, the global financial crisis initiated in 2008, has wiped out years of economic and social progress and exposed structural weaknesses in Europe’s economy (Basarac Sertić et al. 2015; Breman 2011). At the same time it brought the need of undertaking the structural reforms and outlining the long-range priorities of EU development to the authorities’ attention.

The EU comprises of countries with different levels of socio-economic development (Balešentis et al. 2011; Filip 2006, 2009; Fura 2013; Fura and Wang 2015; Halpern 2014; Pudło 2014; Stec et al. 2014; Zuidema and De Roo 2009). The goal of EU cohesion policy is to reduce disparities between individual member states (Barker and Wood 2001; Scherngell and Lata 2013). Therefore, Europe 2020 is another long-term programme for socio-economic growth. Its main objective is to strengthen and develop the economies of all member states, which will be based on knowledge recognised as a major factor determining the modern, international economic competitiveness.

In view of reaching this goal it is essential to monitor changes in levels of development of individual countries and to establish common directions of this development. The strategy should be considered as part of the long-term EU’s economic development plan on its way to increase its international competitiveness. Taking into consideration opinions, analyses and even some calculations made by the researchers of the problem it is possible, more or less, to represent in detail the current state of the progress in implementation of various objectives described in the strategy. However, there is lack of studies that would answer the following questions:

- How to evaluate the implementation of the Europe 2020 objectives in EU member states taking them into consideration not as individual priorities separately but as a whole?
- How each country manages to implement the goals? Which countries are the best and the worst in implementing the objectives?
- How the implementation of the objectives is carried out in the so-called “old member states” (EU-15) and in the countries which acceded to the Union in 2004, 2007 and 2013?
- Which countries can be divided into groups according to the number of implemented objectives?

We hope that the results of our research will help to fill some of the gaps in these emerging areas of interest. We also hope that by joining in the discussion about measuring the progress in meeting Europe 2020 of the EU member states, the results of our research will trigger further analyses in this field and will serve as preliminary studies of the undertaken issues.

This paper is an attempt to compare progress in meeting the Europe 2020 of EU member states from 2009 to 2014. The basis of the assessment is a synthetic measure in dynamic terms, calculated on the basis of the proposed statistical indicators which monitor the implementation of the long-term EU development programme. Taking into consideration the grounds of merit, while building the general synthetic measure, the authors based their work on the zeroed unitarisation method. This method belongs to the so-called multidimensional comparative analysis. These methods make it possible to design synthetic (aggregate) measures based on a number of statistical indicators. Consequently, the results thus obtained are more reliable and objective. The research was conducted dynamically while designing an evaluated synthetic measure for the „object-periods” i.e., countries of UE for the whole period spanning 2009–2014. The static approach, i.e., evaluating of countries for one or several years, treating each as separate periods is often applied in comparative analyses. This approach considerably limits inference.

To achieve the results we are aiming for as well as to answer the questions above, it was necessary to divide the paper into a few sections. The Sect. 2 describes the background for the Lisbon Strategy and the Strategy Europe 2020, and the meaning of these documents for the EU development and for the creation of the most competitive economy in the world. In the following part we present the major indicators, which were employed in the analysis, and which show the realisation of Europe 2020. They are divided into stimulants and destimulants on which basis we tried to determine the values of the synthetic measure. The Sect. 3 presents the statistical appraisal of implementing objectives by the individual EU member states and discusses its outcomes. This section focuses also on maximum, minimum and medium values, coefficient of variation and asymmetry coefficient of the synthetic measure value in years 2009–2014. In this section of the paper the authors ranked the EU member states in terms of a general value of the synthetic measure presenting both rise and fall of the measure for 2014 (in comparison with 2009). The essential part of the analysis was to group EU member states and divide them into groups of high, average–high, average–low and low level of implementing the Europe 2020 objectives. The last section of the paper recapitulates the results and discusses their practical implementation.

## The Europe Strategy: theoretical background

International competitiveness between the United States and the EU has been a subject of research for many years. The competition between these unions in terms of implementing the so-called new economy, based on high technologies, is particularly interesting.

At the end of the 90s it became clear for the European leaders that the economic condition of the EU is relatively smaller than the dynamically developing economy of the United States. In 2000 the average EU’s GDP reached 70% value of this indicator in the United States and it was lower than in the beginning of the 70s. The diagnosis made at the beginning of 2000 by the OECD (2003, 2004) and by the experts from the European Commission clearly stated that the phenomenon of American “new economy” and its accelerated development in IT sector were the results of high innovations which characterise the American economy. Whereas the Union’s economy was described as less dynamic with high unemployment rate, the monopolisation of network industries and low flexibility of labour markets and with low diffusion of advanced technologies. In view of such situation, during the summit in Lisbon in March 2000, the European Council passed a 10-year strategy which aim was to transform the EU into the most dynamic and competitive economy in the world based on knowledge, ability to maintain sustainable economic growth, create more better places of employment and maintain the social cohesion. The stimulation of innovation activities and research and development activities (R&D) (Grimm 2011; Ilbert and Petit 2009) were declared to be the main measures employed to realise this strategic goal.

The Lisbon Strategy, from the substantive perspective, was not a new initiative, as before there had been attempts to introduce various processes in order to recover and reorganise the functioning of the EU. However, its versatility and realisation of comprehensive and integrated attitude to the socio-economic and environmental problems by retaining the principle of subsidiary was innovative (Dearden 2008; Makarovic et al. 2014). The Lisbon Strategy objective was to create the EU’s economy based on knowledge by Lisbon Strategy (2002):

- creating an information society,
- creating a European area for research and innovation,
- creating an environment favourable for developing the innovative entrepreneurships (especially small and medium enterprises),
- integrating financial markets,
- coordinating macroeconomic policies, fiscal consolidation, quality and sustainability of public finances.

The key priorities of the Lisbon Strategy come down to the following issues: innovation, enterprise, synergy (cooperation) and competitiveness (Steurer and Hametner 2013). The Lisbon Strategy’s objectives were very ambitious, but it is a well-known fact that its important goals were not accomplished and Europe 2020 is the latest EU’s economic strategy replacing the Lisbon Strategy. The influence of the global financial crisis on the economic development and objectives and instruments supporting economic development were mainly taken into consideration. The flaws in the implementation of the Lisbon Strategy, redefinition of development challenges, objectives and priorities as well as the evaluation of “technical” aspects of the implementation such as financing, monitoring or social communication were among other issues being at the very heart of the implemented changes in the strategy (The Europe 2020 competitiveness report 2012).

The questions arise whether the ideas included in this new strategy are possible to complete in the prescribed time and whether it should be drawn up and changed. In certain documents (The Europe 2020 competitiveness report 2012, 2014) it is indicated that though the Lisbon Strategy did not meet many objectives its effect on implementing EU development policy should not be evaluated negatively. On the basis of the analysis of these documents, the authors listed its major successes as follows:

- raising awareness of structural reforms as a provision to face up to challenges posed to the EU at the turn of the twenty first century (first and foremost these concerning the EU’s transition to the globally competitive economy based on knowledge),
- stimulating the multilevel governance, i.e. leading the coordinated policies on separate levels of government: union, domestic, regional and local,
- integration of development policy based on the three pillars of sustainability: economic, environmental and social.

It is the progress in the above mentioned areas that will determine the EU’s development potential in the forthcoming years (OECD 2004). In this context, the Strategy 2020 will attempt to continue the coordinated mechanism of managing the EU’s development policy in diverse conditions—among other things due to the different levels of socio-economic development and thus varied development priorities—of the national interests of many member states (Brauers et al. 2012). The challenges that the EU met in 2000 by passing the Lisbon Strategy have become even more urgent as the realisation of the new development strategy is taking place next to the emerging demographic problems (the ageing of Europe’s population is putting at risk the long-term stability of public finances in these countries) which are accompanied by the increasing competition from the rapidly growing markets in Asia (especially India and China) and other countries (e.g. Brazil) as well as the large-scale technological progress (particularly in the ICT area) in the world. If we want to answer the hereinabove questions, it is clear that the modification of strategic regulations and drawing up the new priorities are the essential conditions to increase the competitiveness of EU’s economy (Steurer et al. 2010).

The Europe 2020 has become a part of EU’s functioning procedures; it appears that the central idea of the strategy is to plan development that contains various activity projects.

Europe 2020 is the EU’s ten-year growth strategy puts forward three mutually reinforcing priorities (http://ec.europa.eu/europe2020/index_en.htm):

- Smart growth: developing an economy based on knowledge and innovation.
- Sustainable growth: promoting a more resource efficient, greener and more competitive economy.
- Inclusive growth: fostering a high-employment economy delivering social and territorial cohesion.

These three mutually reinforcing priorities should help the EU and the Member States deliver high levels of employment, productivity and social cohesion The strategy further identifies seven flagship initiatives the EU should take to boost growth and jobs (European Commission 2010):

1. An industrial policy for the globalization era: to improve the business environment, notably for SMEs, and support the development of a strong and sustainable industrial base able to compete globally.
2. A digital agenda for Europe: to accelerate the roll-out of high-speed internet and reap the benefits of a digital single market for households and firms.
3. Innovation Union: to improve framework conditions and access to finance for research and innovation that ensure innovative ideas can be turned into products and services, creating growth and jobs.
4. Youth on the move: to enhance the performance of educational systems and facilitate the entry of young people into the labour market.
5. An agenda for new skills and jobs: to modernize labour markets and empower people by developing their skills throughout the life cycle, with a view to increase labour participation and better match labour supply and demand, including through labour mobility.
6. European platform against poverty: to ensure social and territorial cohesion such that the benefits of growth and jobs are widely shared, and people experiencing poverty and social exclusion are enabled to live in dignity and take an active part in society.
7. Resource-efficient Europe: to help decouple economic growth from the use of resources, support the shift towards a low-carbon economy, increase the use of renewable energy sources, modernize the transport sector and promote energy efficiency.

This strategy should provide a road map for economic recovery and guarantee the EU a strong position in international relations. It is essential that five headline targets are met and have been agreed for the whole EU (European commission 2010; Ruser and Anheier 2014):

- 75% of the population aged 20–64 should be employed,
- 3% of the EU’s GDP should be invested in R&D,
- The “20/20/20” climate/energy targets should be met (including an increase to 30% of emissions reduction if the conditions are right),
- The share of early school leavers should be under 10% and at least 40% of the younger generation should have a tertiary degree,
- 20 million less people should be at risk of poverty.

In order to raise effectiveness in implementing the strategy, the individual member states can support themselves by creating their own policies, such as: action plans and defining goals and short-term, mid-term and long-term actions; preparing qualitative and quantitative measures allowing to make comparisons between the individual member states, between an individual country and the whole Europe or the United States, or any other highly developed country; translate strategic objectives and schedules into national action plans; periodic reviews of the strategy implementation in terms of realisation its objectives and exchanging experiences (Barder et al. 2013; Booth 2013; Ruser and Anheier 2014).

The EU will not accomplish its goals if the individual member states do not pursue them. The Union is a community of great economic potential but with many problems to overcome. It is composed of 28 countries with different levels of socio-economic development. It not only has to deal with the distance between Europe and the United States, but also with the distance existing between “the old” (EU15) and the new member states.

## The attempt to formulate the synthetic measure and the employed method

In the literature there are no precise guidelines, indicators or methods that could be employed to evaluate the progress in implementing the Europe 2020 goals. Ravallion (2011) states that the analyst is free to choose, largely unconstrained by economic or other theories intended to inform measurement practice.

Many authors (Cornescu and Adam 2014; Steurer and Hametner 2013) who are dealing with this issues declare that people voluntarily or involuntarily, are always using indicators when they analyse, forecast and so on. Its importance is given by the fact that indicators are describing a topic of interest, reducing information overload for data users and provides the necessary information for decision-making. The power of the indicators it represents also a weak point because when it is intend to describe a wider topic of interest, the selection of one or more representative indicators is difficult, there is a loss of information risk or manipulation of obtained data. They maintain that, generally speaking, indicators have three main functions. First, they reduce the number of measurements necessary to give a description of a situation (OECD 2003). As such, they are indispensable for measuring progress towards policy objectives (Dalal-Clayton and Krikhaar 2007) and for evaluating the effectiveness of policies (European Commission 2005). Second, indicators simplify the communication of positive and negative developments to politicians, administrators and the public (OECD 2003). Both of these functions rely on the main feature of indicators, i.e. to summarize complexity into a manageable amount of meaningful information that can be understood and interpreted easily. Thirdly, indicators can provide crucial guidance for policy-making (Bossel 1999; UNCSD 2001), in particular regarding the better horizontal integration of policies across sectors, and vertical integration between different levels of government.

There are no indicators that are universally accepted, backed by compelling theory, rigorous in data collection and analysis and influential in policy (Quental et al. 2011). As Bell and Morse (2014) hold, based upon the work of Hezri (2004, 2005) with experience gained over many years working in sustainable development projects spanning many countries, it is possible to conceive a number of possible influences on the use of indicators. They claim that „even if the indicator is very technical and difficult to estimate, if the need is strong enough then it will continue to exist if demand is high. Those demanding the indicators may not necessarily know how it is calculated or its limitations, but as long as the indicator helps them to encapsulate complexity for someone else to understand then it will be of use”.

As some authors point out (Keirstead and Leach 2008) the indicators employed in various analyses should be widely used for systematic performance monitoring, early warning, target setting and other purposes.

Taking into consideration the functions and features that the indicators should fulfil, also concerning the substance and availability of data, the Authors resolved to employ the indicators shared by the EU and published by the Eurostat in order to determine the synthetic measure in meeting Europe 2020 by the member states.

These are the main indicators monitoring the realisation of the Strategy among which we can distinguish the stimulants (S), for which high values are desirable, and destimulants (D), for which low values are recommended. The main indicators have been marked with symbols from X1 to X10.

### Europe 2020 indicators

1. Employment rate by sex, age group 20–64 (%)—(S).
2. Gross domestic expenditure on R&D (GERD)% of GDP—(S).
3. Greenhouse gas emissions, base year 1990 Index (1990 = 100)—(D).
4. Share of renewable energy in gross final energy consumption (%)—(S).
5. Primary energy consumption Million TOE (tonnes of oil equivalent)—(D).
6. Early leavers from education and training by sex% of the population aged 18–24 with at most lower secondary education and not in further education or training—(D).
7. Tertiary educational attainment by sex, age group 30–34 (%)—(S).
8. People at risk of poverty or social exclusion (%)—(D).
9. People living in households with very low work intensity (%)—(D).
10. Severely materially deprived people (%)—(D).

In the paper, in order to evaluate the implementation of the Europe 2020 objectives by the individual member states, the authors employed a synthetic measure in dynamic terms for the period spanning 2009–2014, which was based on the zeroed unitarisation (Kukuła 2000).

The following is a discussion of the methodological assumptions of the proposed method.

Let’s assume that the set data of *n* objects needs to be ordered *O*_*i*_ *(i* = 1, 2, *…* , *n)* which is characterized by *m* features of *X*_*j*_ (*j* = 1, 2, … , *m*). Values of features representing each object is presented as a matrix of observations in the form of:

1 $${\mathbf{X}} = \left[ {\begin{array}{*{20}c} {x_{11} } & {x_{12} } & \cdots & {x_{1m} } \\ {x_{21} } & {x_{22} } & \cdots & {x_{2m} } \\ \vdots & \vdots & \vdots & \vdots \\ {x_{n1} } & {x_{n2} } & \cdots & {x_{nm} } \\ \end{array} } \right]$$

The indicators X1–X10 are represented by different units, so they should first be standardised to determine the synthetic measure A normalization of the variable values is conducted using the following formulas:

2 $$z_{ij} = \frac{{x_{ij} - \mathop {\hbox{min} }\limits_{i} \{ x_{ij} \} }}{{\mathop {\hbox{max} }\limits_{i} \{ x_{ij} \} - \mathop {\hbox{min} }\limits_{i} \{ x_{ij} \} }}\quad{\text{for stimulating factors}}$$

3 $$z_{ij} = \frac{{\mathop {\hbox{max} }\limits_{i} \{ x_{ij} \} - x_{ij} }}{{\mathop {\hbox{max} }\limits_{i} \{ x_{ij} \} - \mathop {\hbox{min} }\limits_{i} \{ x_{ij} \} }}\,\quad{\text{for non-stimulating factors}}$$

The synthetic measure was calculated as an arithmetic mean of the standardized value of variables:

4 $$MS_{i} = \frac{1}{m}\sum\limits_{j = 1}^{m} {z_{ij} }$$

where *MS*_*i*_ synthetic measure, *z*_*ij*_ standardized value of j-th features in i-th object. Synthetic measure *MS*_*i*_ assumes a value between [0;1].

The value of the resulting synthetic measure may serve as the basis for allocating objects (e.g. EU countries) into groups of similar levels of the complex phenomenon being studied. An allocation scheme based on the arithmetic mean and standard deviation of the synthetic measure is also applicable in such circumstances (Stec 2015):

5 $$\begin{array}{*{20}l} {{\text{group}}{\mkern 1mu} {\text{I}}{:}} \hfill & {MS_{i} \ge \acute {s}rMS_{i} + S_{{MS_{i} }} } \hfill & {{\text{high}}{\mkern 1mu} \,{\text{level}}} \hfill \\ {{\text{group}}{\mkern 1mu} {\text{II}}{:}} \hfill & {\acute {s}rMS_{i} + S_{{MS_{i} }} > MS_{{i_{i} }} \ge \acute {s}rMS_{i} } \hfill & {{\text{medium-high}}{\mkern 1mu} \,{\text{level}}} \hfill \\ {{\text{group}}{\mkern 1mu} {\text{III}}{:}} \hfill & {\acute {s} rMS_{i} > MS_{i} \ge \acute {s}rMS_{i} - S_{{MS_{i} }} } \hfill & {{\text{medium-low}}{\mkern 1mu} \,{\text{level}}} \hfill \\ {{\text{group}}{\mkern 1mu} {\text{IV}}{:}} \hfill & {MS_{i} < \acute {s} rMS_{i} - S_{{MS_{i} }} } \hfill & {{\text{low}}{\mkern 1mu} \,{\text{level}}} \hfill \\ \end{array}$$

where $\acute {s}rMS_{i}$ mean value of overall synthetic measure. $S_{{MS_{i} }}$ standard deviation of overall synthetic measure.

## Empirical results

In the next stage of research the values of the synthetic measure for EU countries in 2009–2014 were calculated.

Table 1 illustrates calculated values of the synthetic measure for EU countries in 2009–2014.

**Table 1:** Values of synthetic measures for EU countries in 2009–2014. Source: own calculations based on Eurostat data (www.eurostat.ec.europa.eu)

Country	2009	2010	2011	2012	2013	2014
Belgium	0.624	0.622	0.618	0.628	0.635	0.647
Bulgaria	0.460	0.432	0.414	0.411	0.425	0.490
Czech Republic	0.643	0.649	0.664	0.679	0.693	0.705
Denmark	0.759	0.743	0.751	0.769	0.767	0.787
Germany	0.586	0.583	0.599	0.618	0.625	0.628
Estonia	0.703	0.693	0.724	0.729	0.745	0.725
Ireland	0.564	0.542	0.530	0.537	0.557	0.586
Greece	0.509	0.511	0.466	0.439	0.424	0.449
Spain	0.491	0.482	0.469	0.459	0.465	0.461
France	0.609	0.594	0.604	0.616	0.633	0.632
Croatia	0.504	0.505	0.485	0.478	0.509	0.577
Italy	0.457	0.452	0.440	0.444	0.452	0.476
Cyprus	0.593	0.582	0.585	0.568	0.558	0.574
Latvia	0.579	0.539	0.555	0.600	0.637	0.664
Lithuania	0.649	0.615	0.624	0.651	0.684	0.730
Luxembourg	0.702	0.701	0.704	0.695	0.706	0.722
Hungary	0.500	0.501	0.502	0.505	0.517	0.545
Malta	0.412	0.421	0.437	0.449	0.456	0.467
Netherlands	0.683	0.675	0.686	0.697	0.691	0.693
Austria	0.701	0.700	0.698	0.724	0.731	0.762
Poland	0.554	0.554	0.568	0.583	0.593	0.618
Portugal	0.521	0.529	0.556	0.545	0.540	0.556
Romania	0.446	0.456	0.467	0.470	0.489	0.511
Slovenia	0.696	0.697	0.706	0.716	0.713	0.719
Slovakia	0.595	0.589	0.600	0.610	0.619	0.633
Finland	0.799	0.782	0.787	0.801	0.803	0.794
Sweden	0.866	0.860	0.869	0.886	0.880	0.893
United Kingdom	0.578	0.570	0.596	0.585	0.593	0.624

In 2009–2014, the values of synthetic measure calculated for EU countries generally increase which indicate their progress in meeting Europe 2020. Fluctuations of the synthetic measure for some countries can be observed in 2010 and 2011 which are a result of the global economic crisis from previous years. There are also some differences between the countries in terms of the synthetic measure values. This shows different levels of the Europe 2020 realisation among the individual member states.

The analysis of the arithmetic mean calculated on the basis of the value of the synthetic measure in 2009–2014 (Table 2) presents interesting conclusions concerning the progress in meeting Europe 2020 by the individual member states.

**Table 2:** Values of arithmetic mean for EU countries. Source: own calculations

Years	Arithmetic mean
2009	0.599
2010	0.592
2011	0.597
2012	0.603
2013	0.612
2014	0.631

In 2010–2014 there is a gradual increase of an “average” level of progress in meeting Europe 2020 by EU countries. The decrease in an average value of the synthetic measure in 2010 depicts the unfavourable changes posed by the global crisis. However, the parameter increases gradually in the following years which indicates the progress made by EU countries connected with the implementation of the Strategy. The differentiation of EU countries measured by the coefficient of variance is low and remains within the rate of 19–20%. In all years, the asymmetry coefficient is positive and its value decreases. In the slight majority of EU countries, the level of implementation of the analysed development programme is below the average.

**Fig. 1:**
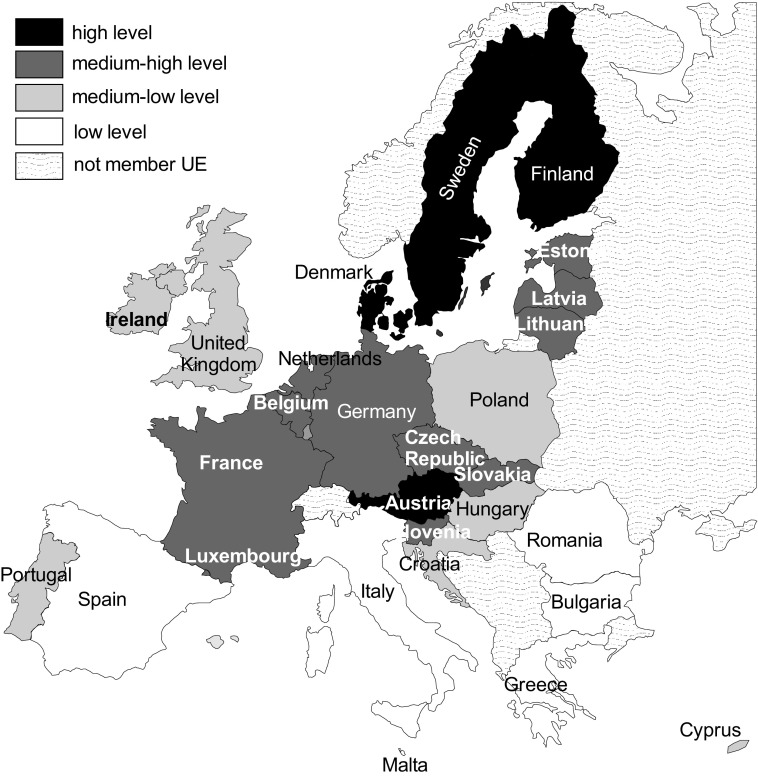
Groups of EU Countries with similar levels of implementation of the Strategy Europe 2020 in 2014. Source: own elaborations

In order to answer which EU countries are on the similar level of the implementation of the Europe 2020, a distribution diagram no. 5 is used and the results of the countries’ classification are presented in the Table 3 and in the Fig. 1.

**Table 3:** Groups of EU countries with similar levels of implementation of the Strategy Europe 2020 in 2009 and 2014. Source: own elaborations

Level	Year	
	2009	2014
High	Sweden, Finland, Denmark	Sweden, Finland, Denmark, Austria
Medium–high	Estonia, Luxembourg, Austria, Slovenia, Netherlands, Lithuania, Czech Republic, Belgium, France	Lithuania, Estonia, Luxembourg, Slovenia, Czech Republic, Netherlands, Latvia, Belgium, Slovakia, France, Germany
Medium–low	Slovakia, Cyprus, Germany, Latvia, United Kingdom, Poland, Portugal, Greece, Croatia, Hungary, Spain	United Kingdom, Poland, Ireland, Croatia, Cyprus, Portugal, Hungary
Low	Bulgaria, Italy, Romania, Malta	Romania, Bulgaria, Italy, Malta, Spain, Greece

High levels of implementation of the Strategy Europe 2020 was, in 2014, showed by Sweden, Finland, Denmark, Austria. These countries can be proposed as the leaders of this development idea. Most countries belong to the group of medium–high (11 countries) and medium–low levels (7 countries).

The group of medium–high level comprises five countries of the “old states” and six countries which acceded to the Union in 2004.

In the group of medium–low level there are more countries which acceded to the Union in 2004. Croatia, which is the latest member state, is also included in this group.

Romania, Bulgaria, Italy, Malta, Spain and Greece are the countries which belong to the group of the lowest level of the implementation of the Strategy Europe 2020. This group includes the countries with the low level of socio-economic development as well as the countries facing serious economic problems.

To show changes in the levels of the strategy implementation by EU countries, the values of general synthetic measures in two critical years, i.e. in 2009 and 2014 were compared, and on their basis EU countries were placed in the ranking (Table 4).

**Table 4:** Positions ranked EU countries in terms of synthetic measure. Source: own calculations

Country	2009	2014	Changing the position in 2014 compared to 2009
Belgium	11	12	−1
Bulgaria	25	24	1
Czech Republic	10	9	1
Denmark	3	3	0
Germany	15	15	0
Estonia	4	6	−2
Ireland	18	18	0
Greece	21	28	−7
Spain	24	27	−3
France	12	14	−2
Croatia	22	19	3
Italy	26	25	1
Cyprus	14	20	−6
Latvia	16	11	5
Lithuania	9	5	4
Luxembourg	5	7	−2
Hungary	23	22	1
Malta	28	26	2
Netherlands	8	10	−2
Austria	6	4	2
Poland	19	17	2
Portugal	20	21	−1
Romania	27	23	4
Slovenia	7	8	−1
Slovakia	13	13	0
Finland	2	2	0
Sweden	1	1	0
United Kingdom	17	16	1

It turned out that in 2014 compared with 2009, 12 EU countries improved their positions (Latvia the most—moved five places upwards, Lithuania and Romania—four places upwards), in six countries the situation did not change, whereas 10 countries fall down in the ranks in terms of the value of the synthetic measure (Greece the most—seven places downwards and Cyprus six places downwards). Invariability of place or its fall in one or two places does not matter for countries which achieve good and very good results in meeting the Strategy Europe 2020 (Sweden, Finland). It is crucial, however, for countries facing serious problems with implementation of the EU development programme. Without their greater involvement as well as significant support, also financial from the EU, these countries may not carry out the objectives of the Strategy Europe 2020.

## Conclusion

The growth of the US economic power, triggered mainly by high investments for research and development and the employment of the most recent technologies, has resulted in the US domination on the global market. The EU, monitoring such situation, has prepared a comprehensive plan of economic development, which initially in the short term and later in the long term was to allow the Europe’s economy meet the challenge of the American economy and as a consequence lead to create the dominant, based on knowledge, economy in the world. The Strategy can be regarded as a response or an attempt to adapt the EU to the processes of globalization while its enlargement of the Central and Eastern European Countries and the Mediterranean countries took place.

It seems reasonable to say that both the US and the EU by means of the mutual competitiveness in the areas of investments, services and trade significantly contribute to the development of the global economy and thus to the betterment of people’s living conditions (Ruser and Anheier 2014).

The EU resolved to complete by 2020 an ambitious development programme based on three main priorities: smart growth, sustainable growth, Inclusive growth. In the paper the authors have made an attempt to statistically evaluate the progress in meeting the Strategy Europe 2020 by the individual EU member states. The indicators proposed by the EU and published by the Eurostat, which were the basis for the evaluation, were also the foundation to determine the synthetic measure in dynamic terms. The evaluation allowed to draw comparisons between EU countries while taking into consideration 10 main indicators, and to group them in terms of the level of progress in meeting the objectives.

The results indicate that in 2014 Sweden, Finland, Denmark and Austria were the leaders in meeting the Strategy Europe 2020 objectives. Most of the EU countries made average progress in the course of implementation of the Europe’s development programme; whereas Romania, Bulgaria, Italy, Malta, Spain and Greece are the countries facing problems in meeting the Strategy Europe 2020. Research has shown that not all EU-15 countries attach the significance in meeting the objectives of the long-term development programme, while among the new EU members (that joined the EU structures after 2004) there are countries for which this programme is a priority.

The delay in the realisation of the objectives in the prescribed time may result in that the development gap between the EU and other global economies (e.g. US or Japan) will not be reduced. It is particularly important to monitor the implementation of Europe 2020 to arrive at the right decisions which will guarantee the attainment of EU’s development strategy.

The conducted research is a preliminary statistical appraisal of the implementation of the Strategy Europe 2020 by the EU countries. It has, however, a great practical significance, hence it provides a general view of the situation in terms of the studied issue; the countries representing the lower progress in meeting the objectives can at least apply for the European funding from the Cohesion Fund or benefit from the experience of the countries with higher level of development. On its basis, one can draw preliminary conclusions from the implementation of the Strategy 2020 objectives and prepare possible directions of changes required for further, successful and effective actions.

It should be emphasized, however, that the results of the implementation will be observed in the long term, therefore, according to the Authors, it is reasonable to conduct further research in the future, while the existing discrepancies between EU countries may become the basis for a specialisation and adoption of specific policy by the authorities of individual member states as well as by the whole EU.

It is hoped that EU internal problems (among other things: UK leaving EU and migration issues) will not impact realization of Strategy 2020 by particular member states.
